# Preliminary study on the effect of parenteral naloxone, alone and in association with calcium gluconate, on bone healing in an ovine "drill hole" model system

**DOI:** 10.1186/1471-2474-8-43

**Published:** 2007-05-22

**Authors:** Lucio Petrizzi, Massimo Mariscoli, Luca Valbonetti, Vincenzo Varasano, Jens D Langhoff, Brigitte Von Rechenberg

**Affiliations:** 1 Department of Veterinary Clinical Sciences – University of Teramo, Italy; 2 Musculoskeletal Research Unit – Vetsuisse University of Zürich, Zürich, Switzerland

## Abstract

**Background:**

Several diseases affect bone healing and physiology. Many drugs that are commonly used in orthopaedics as "analgesics" or anti-inflammatory agents impair bone healing. Stressful conditions are associated with decreased serum osteocalcin concentration. High endorphin levels alter calcium metabolism, blocking the membrane channels by which calcium normally enters cells. The consequent decrease of intracellular calcium impairs the activities of calcium-related enzymes. Naloxone is a pure opioid antagonist. Morphine-induced osteocalcin inhibition was abolished when osteoblasts were incubated with naloxone. Naloxone restored the altered cellular and tissue physiology by removing β-endorphins from specific receptors. However, this is only possible if the circulating Ca concentration is adequate. The aim of the present study was to evaluate the efficacy of parenteral naloxone administration in inducing fast mineralization and callus remodelling in a group of sheep with a standardised bone lesion.

**Methods:**

Twenty ewes were randomly assigned to 4 treatment groups. Group A acted as control, group B received a solution of calcium gluconate, group C a solution of naloxone, and group D a solution of calcium gluconate and naloxone. A transverse hole was drilled in the left metacarpus, including both cortices, then parenteral treatment was administered intramuscularly, daily for four weeks. Healing was evaluated by weekly radiographic examination for eight weeks. For quantitative evaluation, the ratio of the radiographic bone density between the drill area and the adjacent cortical bone was calculated. After eight weeks the sheep were slaughtered and a sample of bone was collected for histopathology

**Results:**

Group D showed a higher radiographic ratio than the other groups. Sheep not treated with naloxone showed a persistently lower ratio in the lateral than the medial cortex (P < 0.01). Histopathology of bone samples showed more caverns and fewer osteoblasts in group D than in the other groups (P ≤ 0.001).

**Conclusion:**

A low-dose parenteral regimen of naloxone enhances mineralization and remodelling of the callus in healing cortical defects in sheep, especially if associated with calcium gluconate.

## Background

Several diseases, including metabolic, neoplastic and infectious conditions, affect bone healing and bone physiology, and many drugs that are commonly used in orthopaedics as "analgesics" or anti-inflammatory agents impair bone healing [1]. The rate of healing is an important factor in the success of treatments for bone diseases: the faster the healing takes place in orthopaedic implants, the less time there is to fatigue the implant system and cause premature loosening or failure [2-4].

Recent studies have considered the effect of metabolic stress on bone physiology [5-7]. Stressful conditions associated with tissue injury are associated with decreased serum osteocalcin concentration [8]. It has also been suggested that high endorphin levels in situations of stress or pain can alter calcium metabolism, blocking the membrane channels by which calcium normally enters cells [9]. The consequent decrease of intracellular calcium impairs the activities of calcium-related enzymes.

In previous studies, opioid receptors were detected in human osteoblasts, and the effects of different opioid agonists on osteocalcin secretion were tested. Osteocalcin synthesis in particular was significantly inhibited by high concentration of morphine and enkephalin [10]. Osteocalcin induces hypocalcemia and its effect on bone is enhancing mineralization and growth [11].

Naloxone is a pure opioid antagonist and blocks each of the opioid receptor types [9]. Morphine-induced osteocalcin inhibition was abolished when osteoblasts were incubated concurrently with naloxone [10]. It was shown that naloxone restores the functional activity of cells and tissue by removing β-endorphins from specific receptors. However, these functions are possible only if the circulating Ca concentration is adequate [12].

It also was concluded that a low-dose parenteral regimen with naloxone can relieve pain by a central pathway mechanism [13]. Naloxone reportedly produces either an increase in clinical pain or a dose-dependent biphasic change: pain intensity increases at high doses and decreases at low doses. Doses of naloxone lower than 1 mg/kg produced a significant decrease in pain intensity in humans and animal models [14-17]. Since analgesia occurs only with low doses, the effect appears to be very specific.

Diagnostic imaging techniques are currently used to evaluate bone healing, particularly radiography but also ultrasonography [18]. A disadvantage of ultrasonography is that the examination is considered operator-dependent; in fracture cases, the area to be examined is relatively complex and large, and the investigation is demanding on the examiner's ability [19].

Creating a bone defect experimentally is a widespread method for evaluating bone healing [19,20]. The evaluation is usually performed with diagnostic imaging systems during the study and/or with histomorphometric evaluation at the end [20]. The time for collecting samples for histomorphometry is usually related to the expected healing time of the defect. In contrast to other models, a "drill hole" model system in a long bone offers the advantage of creating a bilateral cortical defect with minimal trauma; the number and sizes of the defects should be such as to avoid loss of bone stability, to create a protected environment in which the only factors influencing healing are those under investigation.

The aim of this study was to evaluate the efficacy of parenteral naloxone administration in inducing fast mineralization and callus remodelling in a group of sheep with a standardised bone lesion. Calcium gluconate was expected to increase the extracelluar and intracellular concentrations of calcium, improving the activities of calcium-related enzymes in these cells.

## Methods

Twenty Italian Appenninica sheep, non-pregnant females aged 2–4 years (mean 30 ± 5.2 months) and of 48–64 kg body weight (mean 54.2 +/- 4.8 kg), were selected for this study. The animals were all healthy as judged by clinical and hemato-biochemical evaluations. One week before surgery the animals were dewormed with 200 μg/kg of ivermectin intramuscularly (im) (Ivomec ovini, Merial Italia, Milano, Italy). The animal protocol was authorized by the National Government agency responsible for animal welfare and protection (n 001/2001).

### Pre-surgical evaluation

Before surgery (T0), every animal was subjected to clinical orthopaedic evaluation and dorso-palmar and latero-medial radiographic views of the left metacarpus were obtained (90 KW, 100 mA, 0.26 s).

### Surgery

General anaesthesia was induced with a single dose of 0.2 mg/kg xylazine chloride (Rompun, Bayer, AG Leverkusen, Germany) and 10 mg/kg ketamine chloride (Ketavet 100, Intervet Italia srl, Milano, Italy), given intramuscularly. Thereafter, the sheep were intubated and anaesthesia was maintained using isofluorane (Isoba, Schering Plough Animal Health, Harefield, UK) in oxygen. A ruminal rino-gastric tube was placed to avoid tympany during surgery. The sheep were placed in lateral recumbency and the proximal third of the left metacarpal bone was prepared for aseptic surgery.

A 2 cm vertical incision was made through the skin and down to the bone at the proximo-medial aspect of the left metacarpal bone, about 4 cm distal to the carpo-metacarpal joint. Then a 4 mm wide hole was drilled transversely through both the medial and the lateral cortices of the bone; the periosteum was removed with the drill bit from both sides of the metacarpus. The bone debris was flushed out of the hole with sterile saline. The skin was closed with simple interrupted sutures.

### Treatment

After surgery the sheep were randomly assigned to one of four treatment groups (A, B, C, D), each comprising five animals. The treatment in every group was administered im, over 24 h, for four weeks (T1-T5). Group A (control) received 0.1 ml/kg of 0.9% NaCl, group B (calcium group) 0.1 ml/kg of 20% calcium gluconate (Calcio ph, Fatro, Ozzano Emilia, Italy), group C (naloxone group) 0.1 ml/kg of 0.02% naloxone (Naloxone hydrochloride, Dyosint, Amsterdam, Holland) and group D (calcium-naloxone group) 0.1 ml/kg of solution containing 0.02% naloxone and 20% calcium gluconate. A 4-week treatment was used because bone is mostly resorbed during the first 3–4 weeks and little new bone is produced in a 4 mm cortical defect [19]. The naloxone dosage chosen (0.02 mg/kg) is consistent with that reported to have an analgesic effect [14-17].

All the sheep were treated with amoxicillin (20 mg/kg q 12 h, im) (Clamoxyl RTU, Pfizer Italia, Roma, Italy) for three days after surgery. They were allowed to recover in large stalls with immediate ambulation; no bandage was applied over the operated limb.

### Post-operative evaluation

The animals were evaluated clinically every day to determine general condition and lameness. Lameness was evaluated by observing each animal moving freely in the stall and was scored on a 4 degree scale. Bone healing was followed in each animal by radiographic evaluation immediately after surgery (T1) and every week thereafter for 8 weeks (T2-T9) using the same technique as at T0. Thereafter, every radiographic picture was scanned (Duoscan T1200 flatbed scanner, Agfa Italia, Milano, Italy) and processed using graphic software (Corel Photo-Paint^® ^CorelDraw, Microsoft Italia, Segrate, Italy). A total area 10500 pixels (70 × 150 h) was selected, over both the medial and the lateral cortices at the site of drilling and over the normal adjacent bone. A histogram of the area was then obtained by measuring the number of pixels with each of the 256 levels of grey, ranging from white (255) to black (0). The mean level of grey, with standard deviation, was obtained for each area. Only the dorso-palmar views could be evaluated adequately with the computer-assisted system because the superimposition of the lateral and medial cortical defects was incomplete and variable, and it was impossible to evaluate the healing of the medial and lateral cortices separately in the latero-medial views.

**Figure 1:**
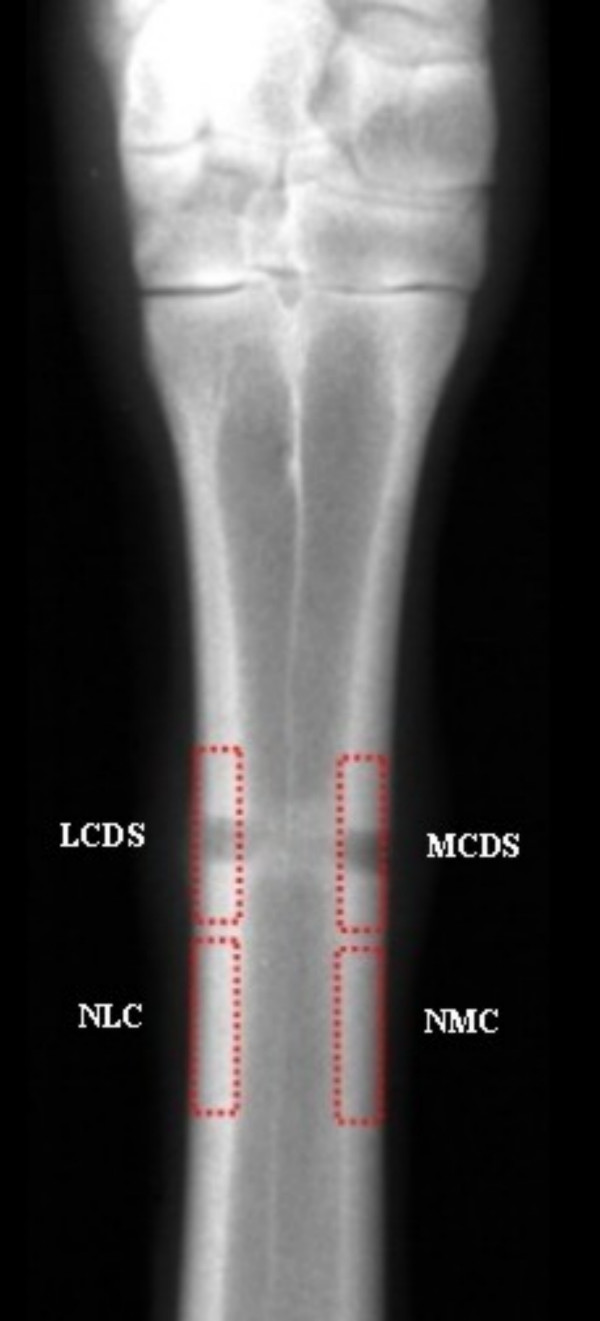
Dorso-palmar radiograph of the metacarpus of one sheep (A5) after surgery (T1): selection of an area of 10500 pixels (70 × 150 h), indicated by the red dotted line, over the medial and lateral cortices, at the drill site (MCDS, LCDS) and on the adjacent normal bone (NMC, NLC), to be processed by graphic software (Corel Photo-Paint, CorelDraw) to obtain the histogram for each site. The LCDS/NLC and MCDS/NMC ratios were calculated to evaluate bone healing at different times.

For each dorso-palmar radiograph, four histograms were obtained: normal medial cortex (NMC), normal lateral cortex (NLC), medial cortex (MCDS) and lateral cortex at the drilling site (LCDS) (Figure 1). The LCDS/NLC and MCDS/NMC ratios were calculated to evaluate the bone remodelling. These ratios were also used for statistical tests.

### Histopathological evaluation

After eight weeks (T9) all the sheep were slaughtered and a sample of bone, 2 cm long, was cut transversely from the left metacarpal directly at the surgical drilling site. The slice of bone obtained was then divided axially into two parts, lateral and medial. All the samples were immediately stored in 4% buffered formalin at 4°C until further processing as non-decalcified bone specimens. Before cutting the final slices of bone the samples were embedded in acrylic resin [21]. Briefly, after a series of steps of alcohol dehydration and defatting with xylene, the specimens were infiltrated and embedded in methylmethacrylate (HistoDur^®^, Leica, Switzerland). The slices were cut perpendicularly to the drill hole axis; only cortical slices were selected for histopathological examination. Histopathology was used to evaluate mineralization and callus remodelling; as the surrounding bone is highly involved in callus remodelling, only transverse sections could show all the cortical bone surrounding the defect.

Three kinds of evaluation were performed: qualitative, quantitative and semi-quantitative. The qualitative and the semiquantitative evaluations were performed using 5 μm sections prepared with a microtome (Leica SP 1400, Wetzlar, Germany) and stained with Toluidine blue or von Kossa/MacNeal. The slides were examined with a light microscope (Leica DMR) at different magnifications (10×, 20× and 40×). For the semiquantitative evaluation the following variables were considered: presence of caverns (remodelling space) in the bone surrounding the drill hole; numbers of osteoblasts and osteoclasts; and the appearance of mesenchymal cells and macrophages and the presence of fibrous tissue or cartilage inside the drill hole. Each evaluation was scored on a 4-point scale (0–3) of increasing quantity/number (Table 1).

For quantitative evaluation, ground sections were prepared. Bone samples were cut using a sawing microtome (Leica SP 1600, Wetzlar, Germany) and mounted on acropal plastic slides (Perspex GS opal acrylic glass 1013, Wachendorf AG, Switzerland). After grinding and polishing to approximately 30–40 μm (Struers, Birmensdorf, Switzerland), they were surface-stained with toluidine blue. New bone formation, parent bone and fibrous tissue within and around the "drill hole" were quantified using an image analysis system (LeicaQWin, Leica Imaging Systems, Cambrige, England) coupled to a light microscope (5.8-fold magnification, Leica M420) with a digital camera (Leica DC200, Wetzlar, Germany). The digitalized resolution was 1 pixel per 8.93 μm. The optical reference was a standardized area of 49.9 mm^2 ^(corresponding to an 8 mm diameter) overlying the defect. Bone area and void area were encircled on the computer by a person blinded to the experimental groups. The amounts of new bone, parent bone and fibrous tissue were expressed as fractions of the total measured area.

### Statistics

To evaluate the efficacy of the proposed treatments, the results from each group, including the radiographic ratios and histopathology, were compared statistically. Normal distribution of data was previously assessed by means of Kolmogorov-Smirnov test (SPSS 13.0 for Windows SPSS inc).

The radiographic ratio data appeared normally distributed, therefore parametric statistic was applied; data were evaluated by multiple pairwise comparisons for One-Way layout design tests such as ANOVA and the Tukey Test (Koichi Yoshioca^® ^KyPlot version 2.0); repeated measures ANOVA was also performed using SPSS Manova (SPSS 13.0 for Windows SPSS inc). The correlation between the radiographic ratios of the medial and lateral cortices was assessed by linear regression and Pearson Product Moment Correlation (Microsoft^® ^Excel 2002).

The histopathological evaluation results were estimated by two nonparametric tests for unpaired data, the Kruskal-Wallis Test and the Wilcoxon Rank Sum Test (Mann-Whitney U Test) (Koichi Yoshioca^® ^KyPlot version 2.0).

Differences were considered significant at P values < 0.05.

## Results

### Surgery

All the sheep recovered from anaesthesia; the surgical procedure was simple and no complications occurred. After surgery only mild lameness was observed. Some swelling developed over the surgical site in all the animals but resolved within five days. By the second day after surgery, all the sheep were weight-bearing on the operated limb.

One sheep in group C (naloxone) died on day 25 for causes unrelated to the experimental protocol (grain overload and metabolic acidosis). Nevertheless a specimen of bone was collected from the left metacarpus at the surgical site. It was used for morphological evaluation like the other samples, but was excluded from the statistical evaluation.

### Radiographic ratio

In all the animals the ratios for the lateral and medial cortices were taken as 1 at T0. At T1, immediately after surgery, the LCDS/NLC and MCDS/NMC ratios decreased to a mean value of 0.91, then progressively increased in all groups. The mean value increased to 0.97 at T5, decreased to 0.95 at T5-T6 and then rose again until T9. The maximum value of 0.97 was reached again at T9.

The curves of the mean ratios for the lateral and medial cortices differed. In the lateral cortex, the mean ratio rose from 0.90 at T1 to reach a maximum of 0.95 at T5; this decreased slightly over the subsequent measurements. The curve for the medial cortex rose from 0.92 at T1 to 0.99 at T5, then decreased slightly, then increased further to 0.99 at T9.

**Figure 2:**
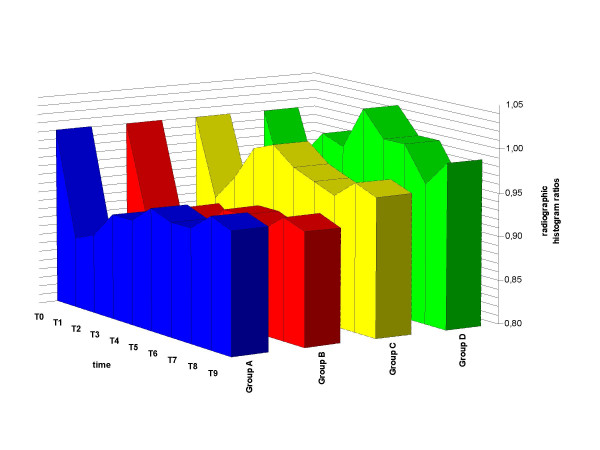
Radiographic histogram ratios: mean values at each time point in each treatment group.

The overall curve rose slowly from 0.88 (T1) to 0.94 (T9) in group A, and from 0.90 (T1) to 0.93 (T9) in group B, while for groups C and D the curve was biphasic, rising sharply from 0.91 (T1) to 0.99 (T4) and then again from 0.95 (T7) to 0.96 (T9) in group C and from 0.92 (T1) to 1.03 (T5) and again from 0.96 (T8) to 0.99 (T9) in group D (Table 2, Figure 2).

**Figure 3:**
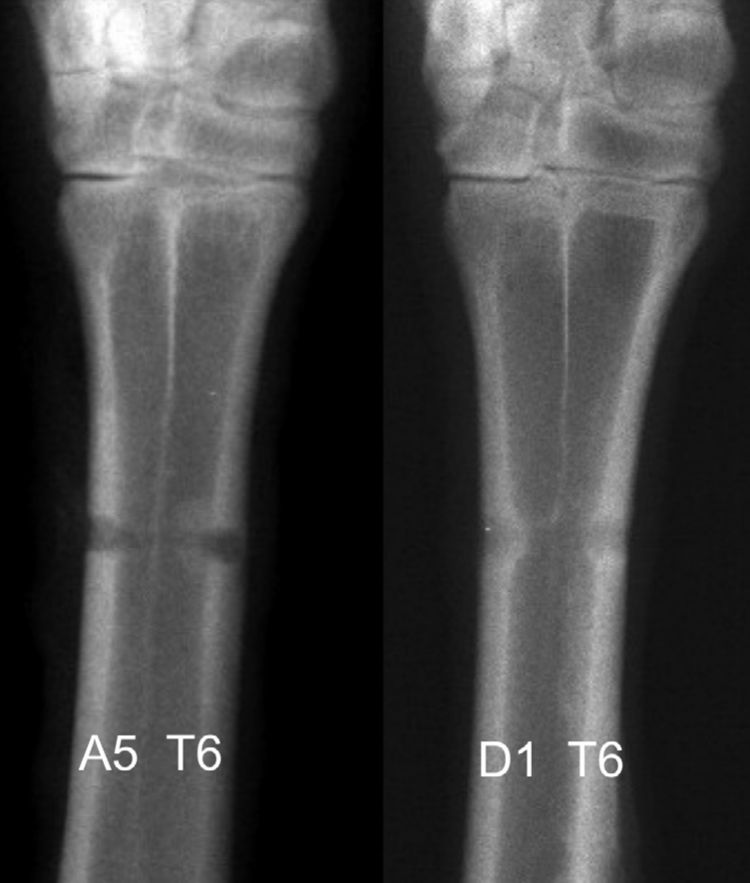
Comparison of a dorsopalmar radiography of the metacarpus of a sheep from Group A (control) and one of Group D (naloxone and calcium gluconate) at T6. The difference in radiodensity at the drill hole site is evident and is statistically significant.

The ratios had higher values in group D than in any other group; the difference was significantly greater at T3 and T6 in group D than in groups A and B (P < 0.01) (Figure 3).

In groups A and B there was a persistently and significantly (P < 0.01) lower ratio for the lateral cortex (LCDS/NLC) than for the medial (MCDS/NMC).

### Histopathology

#### Qualitative and semiquantitative evaluation

In all the samples the drill holes showed some degree of ossification, although none was completely closed. New, woven bone was established at the periphery while fibrous tissue was still present in the centre; no cartilage or other signs of enchondral ossification were detected. Osteoblasts and osteoclasts were aligned along the lamellae of the new bone, and caverns were found in the parent bone surrounding the drill hole. In the samples from group D the number of caverns (remodelling space) was significantly higher while the number of osteoblasts was significantly lower than in any other group (P < 0.001), indicating an advanced stage of remodelling.

**Figure 4:**
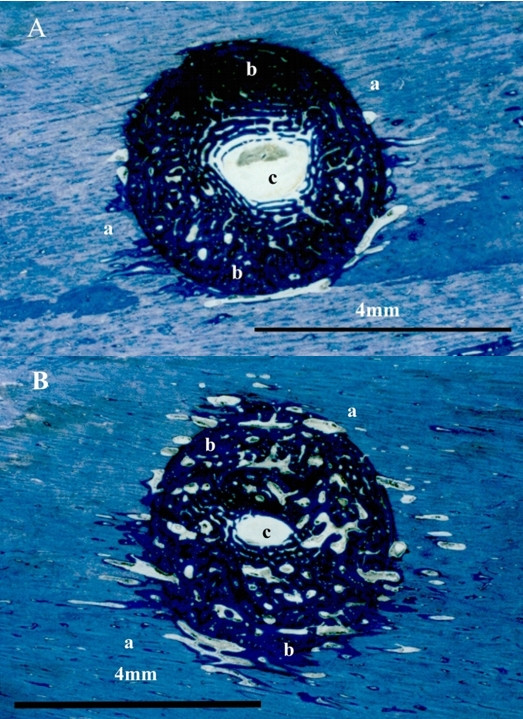
Semiquantitave histopathological evaluation of the samples obtained after eight weeks from surgery. (A) Microphotograph (10 ×; Toluidine Blue) of a slide obtained from a sheep of group A (Control); (B) Microphotograph (10 ×; Toluidine Blue) of a slide obtained from a sheep of group D (Naloxon and Calcium gluconate). Comparing the images is particularly evident the diffence in the number and area of the caverns surrounding the "drill hole" a. Parent bone; b. New bone; c. Fibrous tissue.

In group A (control), thin sections revealed only a few caverns (grade 0.7), many osteoblasts inside the bone callus (grade 2), and a moderate quantity of fibrous tissue (grade 1.2) (Figure 4A). In groups B (Ca) and C (Nal), the values did not differ much from the control group except for the number of osteoblasts (grade 1.375 in group C). Group D specimens showed many caverns (grade 2) around the drill hole, a moderate quantity of fibrous tissue (grade 0.9), and fewer osteoblasts inside the bone callus (grade 1) (Figure 4B). The numbers of macrophages, osteoclasts and mesenchymal cells were similar in all groups (Table 3). Statistically, the most significant differences among the groups were in the number of remodelling cavities and osteoblasts.

The group C sheep that died on day 25 showed absence of caverns (0), a moderate number of osteoblasts and osteoclasts (1) and much fibrous tissue (3) inside the drill hole.

#### Quantitative evaluation

Computerized quantitative analysis of the bone samples obtained eight weeks after drilling the hole and four weeks after termination of the treatment showed no statistically significant difference in the percentages of new bone, parent bone or fibrous tissue among the groups or between the cortices. However, the median percentage of fibrous tissue was higher in groups A (75.0) and B (78.9) than in groups C (67.6) and D (71.0) (P > 0.05). The median percentage of parent bone was higher in groups A (6.84), B (8.45) and C (8.1) than in group D (5.0) (P > 0.05). The median percentage of new bone was lower in groups A (18.6) and B (13.88) than in groups C (25.63) and D (22.29) (P > 0.05).

## Discussion

In this study, a low dose parenteral regimen of naloxone was found to enhance mineralization and remodelling of 4 mm cortical defects of the metacarpal bones of sheep, especially if the treatment was combined with calcium gluconate administration.

The "drill hole" model proved a good system for evaluating bone healing [1,19,20]. The surgery was easy to perform and avoided the need for, and the influence of, fixating devices to stabilize the fracture. Furthermore, if the drill hole is perpendicular to the axis of the limb, as in our case, both medial and lateral cortices can be evaluated to detect differences in callus formation. The diameter of the drill hole (4 mm) corresponds to what is considered a critical-sized bone defect, which does not heal alone, at least in the time period chosen for these experiments [1,2,19,20]. The radiographic histogram ratio was a reliable dynamic indicator of bone healing, with a low standard deviation for each mean value.

To investigate the persistence of the effect of naloxone on bone healing and remodelling, sample collection for histopathology was delayed for 4 weeks after the parenteral treatments of the sheep had ended. The number of sheep, low for ethical and financial reasons, did not permit bone samples to be collected at different time points after termination of the treatment. For these reasons, all the sheep were slaughtered at the same time, 8 weeks after the beginning of the experiment and 4 weeks after the end of the various treatment modalities. The absence of cartilage or any other sign of endochondral ossification from any of the samples examined could be due to the lack of micromotion in the drill hole model, and/or to an efficient blood supply and high oxygen tension [22].

The greatest increase in radiographic ratio after surgery was found in the sheep treated with calcium gluconate and naloxone (group D) (Figure 3). In the group treated with naloxone alone (C), the increase was higher than in the other groups (A and B), but not as high as in group D.

The differences between the radiographic ratios at T3 and T6 were statistically significant for group D, and also for groups A and B. This means that two weeks after the beginning (T3) and one week after the end (T6) of treatment, the drill holes were significantly more radiodense in the sheep receiving naloxone together with calcium gluconate than in any other group. According to our results, this effect was particularly evident two weeks after the beginning of treatment and lasted for about one week after the end. These findings support the hypothesis that a low-dose parenteral naloxone regimen enhances the mineralization of the callus, especially when combined with calcium gluconate administration. As shown in previous studies, the calcium-related activities of the cells could be increased because the intracellular calcium is increased by the action of naloxone on the membrane pumps [9]. Extracellular calcium is also thought to enhance the increase in naloxone-induced intracellular calcium in a dose-dependent manner [23]. In our study the sheep receiving both calcium and naloxone had the highest ratios.

In the animals treated with naloxone alone (group C), or with naloxone together with calcium gluconate (group D), there was no difference in the increase of radiodensity between the lateral and medial cortical defects. In the other groups (A and B) the lateral cortex healed significantly more slowly than the medial. The different healing pattern might be caused by the medial access of surgery, irritating the periosteum and surrounding tissue and leading to an activated tissue response; in this case the effect would have been evident in every group. The difference in healing pattern may also be attributed to mechanical factors or different vascular supplies [24-26]. It may be assumed that the limbs of the sheep receiving the low-dose naloxone regimen had better and more balanced weight-bearing and/or better perfusion, enhancing the healing of the lateral cortex [14-17]. Decreased pain perception after surgery and better subsequent mechanical stimulation through increased weight-bearing by the limbs may improve vascularisation and thus the healing of the bone. However, it is difficult for the observer to judge recognition and perception of pain in sheep [27], and this could explain the lack of evident difference in clinical response among the treatment groups after surgery, despite the administration to two groups (C and D) of a naloxone regimen that had shown an analgesic effect in previous studies [10,13-17]. The clinical observation of weight-bearing proved inadequate for pain assessment in sheep after surgery. To answer these questions, more objective data should be obtained in future studies for either mechanical load (treadmill) or vascularisation (immunohistochemistry) after naloxone application.

The analgesic properties of naloxone could reduce pain perception from the operated limb and improve its use [14-17,28]; previous studies [24-26] have demonstrated that early mechanical load over a fractured limb improves bone healing and quality of callus, enhancing microvascularization [25]. Several experimental studies on animal models and human patients have confirmed the pain-relieving effect of low-dose naloxone (lower than 1 mg/kg) [14-17,28], but no data are available in the literature on the use of naloxone for fracture patients.

Qualitative histopathological examination of the samples showed more bone caverns (P < 0.001) and lower cellularity around the drill hole in group D; both findings are compatible with a more advanced stage of bone healing and remodelling. No statistically significant differences could be demonstrated between groups by quantitative evaluation. Nevertheless, in the samples from groups C and D, both treated with naloxone, there was evidence of more new bone and less fibrous tissue and parent bone than in the other groups. The lack of significance could be related to the low numbers of animals per group, the high individual variability and/or the relatively long time period between cessation of treatment and sacrifice.

A number of limitations must be noted in this study. Primarily we have to consider the low number of animals, chosen to satisfy ethical concerns and limited by the law. Nonetheless, we obtained statistically significant differences in radiographic ratios and qualitative histopathological evaluation.

On the basis of our results, a histomorphological evaluation of bone samples collected 2 weeks after the beginning and one week after the cessation of a 4-week treatment with naloxone and calcium gluconate would be interesting; these are the time points at which statistically significant differences in the radiographic ratios were detected. Such a study would confirm the enhancement of the mineralization of the cortical defect

## Conclusion

In conclusion, this study shows that a low-dose parenteral regimen of naloxone, especially if associated with calcium gluconate, aids mineralization and enhances earlier remodelling of the cortical bone in a 4 mm drill hole model system. Further studies are needed to establish the effects of frequency and overall length of the treatment. A segmental osteotomy model could also permit the effects of naloxone alone and in association with calcium gluconate to be evaluated in a mechanically unstable environment.

## Competing interests

The author(s) declare that they have no competing interests.

## Authors' contributions

LP conceived, supervised and coordinated the study and its design, and drafted the manuscript. MM participated in the design of the study, statistical analysis and revision of the manuscript. LV performed the pre- and post-surgical clinical and radiographic evaluations, processed the radiographic images and calculated the histograms. VV performed the surgery, supervised the postoperative treatment of the animals and was in charge of their welfare throughout the protocol to euthanasia and the collection of histopathology samples. JDL performed the histopathological studies and helped with the statistical analysis. BVR supervised and coordinated the histopathology, and revised the statistical analysis and the manuscript. All authors read and approved the final manuscript.

## Pre-publication history

The pre-publication history for this paper can be accessed here:



## Figures and Tables

**Table 1:** Correspondence of the score (0–3) for the histological findings for each parameter considered during semiquantitative histopathological evaluation.

Grade	Surrounding Cavities	Macrophages Mesenchymal Cells	Osteoblasts	Osteoclasts	Fibrous Tissue	Cartilage
0	None	None	None	None	None	None
1	Occupying less than 25% of the bone surrounding the drill hole	One or two cells per field of view within the drill hole	Less than 25% of the field of view within the drill hole occupied by cavities lined with osteoblasts	One or two osteoclasts per field of view within the drill hole	Less than 25% of the drill hole occupied by fibrous tissue	Less than 25% of the drill hole occupied by cartilage
2	Occupying up to 50% of the bone surrounding the drill hole	Up to five cells per field of view within the drill hole	Up to the 50 % of the field of view occupied by cavities lined with osteoblasts	Up to five osteoclasts per field of view within the drill hole	Up to 50% of the drill hole occupied by fibrous tissue	Up to 50% of the drill hole occupied by cartilage
3	Occupying more than 50% of the bone surrounding the drill hole	More than five cells per field of view within the drill hole	More than 50% of the field of view occupied by cavities lined with osteoblasts	More than five osteoclasts per field of view within the drill hole	More than 50% of the drill hole occupied by fibrous tissue	More than 50% of the drill hole occupied by cartilage

The slides were examined with a light microscope (Leica DMR) at different magnifications (10×, 20× and 40×).

**Table 2:** Radiographic histogram ratios: mean values at each time point in each treatment group.

**Time**	**T0**	**T1**	**T2**	**T3**	**T4**	**T5**	**T6**	**T7**	**T8**	**T9**
**Group A**										

**Mean**	1.00	0.88	0.89	0.92	0.92	0.94	0.93	0.93	0.95	0.94
**St. Dev.**	0.00	0.04	0.04	0.03	0.05	0.03	0.05	0.06	0.06	0.09

**Group B**										

**Mean**	1.00	0.90	0.91	0.92	0.90	0.93	0.93	0.92	0.94	0.93
**St. Dev.**	0.00	0.05	0.02	0.04	0.08	0.05	0.04	0.05	0.04	0.05

**Group C**										

**Mean**	1.00	0.91	0.94	0.98	0.99	0.97	0.96	0.95	0.97	0.96
**St. Dev.**	0.00	0.09	0.05	0.05	0.04	0.07	0.04	0.08	0.06	0.04

**Group D**										

**Mean**	1.00	0.92	0.96	0.99	0.98	1.03	1.00	1.00	0.96	0.99
**St. Dev.**	0.00	0.05	0.06	0.04	0.06	0.11	0.04	0.11	0.10	0.07

**Table 3:** Semiquantitative evaluation scores of the samples obtained from each cortex of every animal.

**Score after semiquantitative evaluation**							
**Group**		**Cavities**	**Macrophages**	**Osteoblasts**	**Osteoclasts**	**Fibrous t.**	**Mesen. Cel.**

**A**	**Mean**	0.70	0.50	2.00	1.20	1.20	0.40
	**St. dev.**	0.82	0.71	0.00	0.42	0.63	0.70
**B**	**Mean**	0.00	0.20	2.00	1.00	1.70	0.60
	**St. dev.**	0.00	0.42	0.00	0.00	0.67	0.52
**C**	**Mean**	0.63	0.38	1.38	0.75	1.63	0.88
	**St. dev.**	0.92	0.52	0.52	0.46	0.74	0.35
**D**	**Mean**	2.00	0.10	1.00	0.70	0.90	0.40
	**St. dev.**	0.94	0.32	0.00	0.48	0.74	0.52

The parameters evaluated were number of remodelling cavities, presence of macrophages, number of osteoblasts and osteoclasts, quantity of fibrous tissue and presence of mesenchymal cells. Evaluation scores refer to 4 grades (0–3) of increasing quantity/number.
